# Identification of a New Mutation in *RSK2*, the Gene for Coffin–Lowry Syndrome (CLS), in Two Related Patients with Mild and Atypical Phenotypes

**DOI:** 10.3390/brainsci11081105

**Published:** 2021-08-22

**Authors:** Mariateresa Di Stazio, Stefania Bigoni, Nicola Iuso, Josef Vuch, Rita Selvatici, Sheila Ulivi, Pio Adamo d’Adamo

**Affiliations:** 1 Institute for Maternal and Child Health-IRCCS “Burlo Garofolo”, 34127 Trieste, Italy; nicolaiuso94@gmail.com (N.I.); josef.vuch@alice.it (J.V.); sheila.ulivi@burlo.trieste.it (S.U.); pio.dadamo@burlo.trieste.it (P.A.d.); 2 Medical Genetic Unit, Department of Mother and Child, Ferrara University Hospital, 44121 Ferrara, Italy; stefania.bigoni@unife.it; 3 Medical Genetics Unit, Department of Medical Sciences, University of Ferrara, 44121 Ferrara, Italy; rita.selvatici@unife.it; 4 Medical, Surgical and Health Sciences Department, University of Trieste, 34127 Trieste, Italy

**Keywords:** *RSK2* gene, Coffin–Lowry syndrome, intellectual disability, kinase assay, functional assay

## Abstract

Background: Coffin–Lowry syndrome (CLS) is a syndromic form of X-linked intellectual disability, in which specific associated facial, hand, and skeletal abnormalities are diagnostic features. Methods: In the present study, an unreported missense genetic variant of the ribosomal S6 kinase 2 (*RSK2*) gene has been identified, by next-generation sequencing, in two related males with two different phenotypes of intellectual disability (ID) and peculiar facial dysmorphisms. We performed functional studies on this variant and another one, already reported in the literature, involving the same amino acid residue but, to date, without an efficient characterization. Results: Our study demonstrated that the two variants involving residue 189 significantly impaired its kinase activity. Conclusions: We detected a loss-of-function *RSK2* mutation with loss in kinase activity in a three-generation family with an X-linked ID.

## 1. Introduction

Coffin–Lowry syndrome (CLS) (MIM 303600) is a rare X-linked dominant disorder, first described by Grange S. Coffin in 1966 and characterized by intellectual disability (ID) and peculiar facial dysmorphisms, hands, and skeletal malformations [1].

In 1971, Robert Brian Lowry described in a three-generation family another syndrome with intellectual disability, small stature, hypotonia and facies characterized by hypertelorism, anteverted nares and a prominent frontal region [2,3]. Temtamy et al., in 1975, asserted that these conditions constituted one single disease called, since then, Coffin–Lowry syndrome [3,4].

In male patients with CLS, moderate-to-severe intellectual disability, abnormal gait, skeletal abnormalities and characteristic facial changes are the rule. Moreover, they typically have hypotonia, delayed closure of the anterior fontanel, facial dysmorphisms, short stature, tapering, hyperextensible fingers, and progressive skeletal deformities, whereas carrier females may show a typical CLS phenotype but are commonly mildly affected [1,5].

Coffin–Lowry syndrome is caused by mutations in the *RSK2* (RPS6KA3) gene located at Xp22.2, which codes a member of the ribosomal S6 protein kinase family. *RSK2* is a growth factor-regulated serine-threonine protein kinase of 740 amino acids (90 kDa) that acts at the distal end of the ras-mitogen-activated protein kinase (MAPK) signaling pathway [6]. The protein shows a strong expression in humans, such as in the embryonic and adult mouse brain, in crucial regions for cognitive function and learning, such as synaptic activity, neocortex, hippocampus, and Purkinje cells. RSK proteins are composed of two functional kinase catalytic domains: the N-terminal kinase domain belongs to the AGC kinase family, and the C-terminal kinase domain belongs to the CamK family linked by a region of 100-amino-ac. RSK proteins are directly phosphorylated and activated by ERK1/2 in response to growth factors, neurotransmitters, and many polypeptide hormones [7]. A range of mutations has previously been described in CLS patients, including truncating and missense mutations. The variations are spread along the gene’s length, although most are located in one of the two kinase domains [8]. The truncating mutations have been found in about 80% of families, and in general, these have been associated with more severe physical and intellectual impairment [3,9,10], while approximately 20% of modifications are missense mutations. The high conservation of amino acids in the two kinase domains supports the hypothesis of their essential functional and structural involvement; therefore, missense mutations are critical for the catalytic function of RSK2, for the site ERK docking and folding, and the binding sites of the ATP.

In the present study, we reported a novel mutation in the *RSK2* gene present in two related patients with different phenotypes characterized by ID and peculiar facial dysmorphisms.

Furthermore, we analyzed the mutated protein expression and function to evaluate the genetic variant’s pathogenicity and performed the same functional studies on an already reported mutation involving the same amino acid residue.

## 2. Results

### 2.1. Clinics

**Figure 1:**
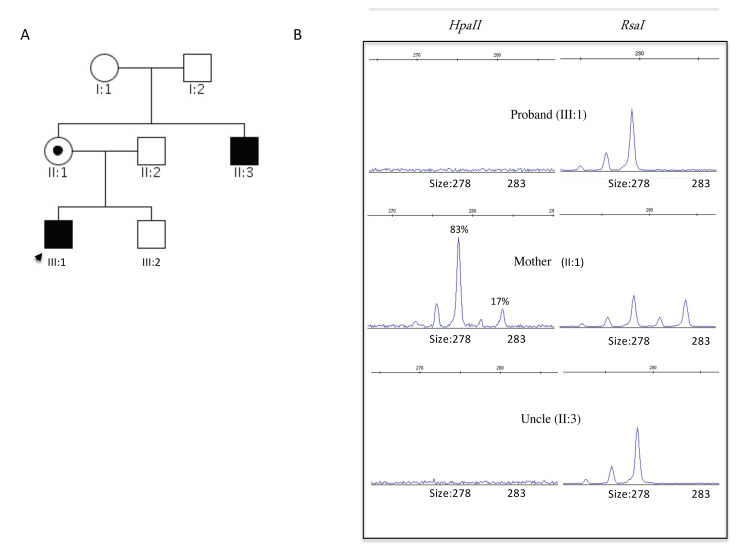
(**A**) The three-generation pedigree for the Italian family affected by intellectual disability. The arrow indicates the proband. (**B**) Analysis of the human polymorphic marker in genomic DNA (from blood cells) digested with HpaII and RsaI enzymes.

Here we report a Caucasian family with recurrence of ID in two related male subjects (III:1, II:3), suggestive of an X-linked inheritance (Figure 1).

### 2.2. Case 1 (III:1): The Proband

The proband (patient III:1) was referred to the Medical Genetic Unit of Ferrara University Hospital for dysmorphology and genetic evaluation in 2006 at the age of 10. He was the first son of a couple of non-consanguineous parents and was born at term by spontaneous delivery after an uneventful pregnancy. Hypotonia, late closure of the anterior fontanelle, and right amblyopia were reported in infancy along with mild psychomotor development delay. The evaluation of the IQ score performed at age 13 years revealed a mild intellectual disability (IQ of 60). At the next re-evaluation at the age of 18, the impairment was severe (IQ of 34). Both the receptive and the expressive language were reported as delayed. He also developed behavioral problems during growth, mainly characterized by *attention deficit*, hyperactivity disorder and impulsiveness/aggressiveness episodes with peers.

**Figure 2:**
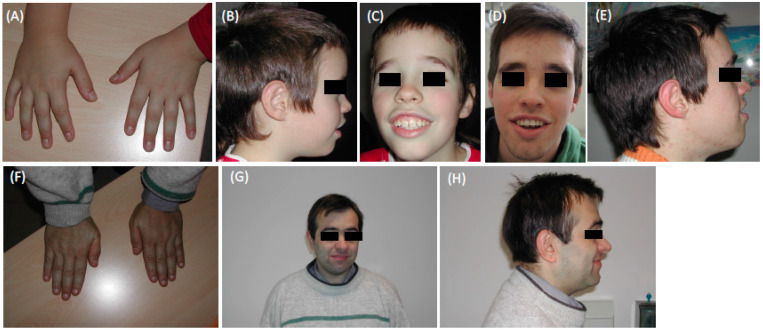
(**A**–**C**) Patient (III:1) 9 years old, (**A**) short hands with relatively long fingers, mild joint stiffness; (**D**,**E**) patient 17 years old. Note the course of dysmorphic features with age. Note high forehead, bitemporal narrowing, long face, telecanthus, down-slanting palpebral fissures, malar hypoplasia, wide mouth with the thick and everted upper and lower lip. (**F**–**H**) Uncle (II:3), note brachycephaly, round face, high forehead, hypertelorism, arched eyebrows with synophrys, deviated nasal septum, bulbous nasal tip with the low hanging columella, lower lip not thick and only slightly everted. (All photographs reproduced with permission).

The main signs at the last dysmorphology evaluation (at the age of 20 y) have been: stature 162 cm (≤3rd centile), OFC (orbifrontal cortex) 59.5 cm (>97th centile), dolichocephaly, facial dysmorphisms (high forehead, bitemporal narrowing, long face, telecanthus, down-slanting palpebral fissures, malar hypoplasia, wide mouth with thick and everted upper and lower lip, total hand length 18 cm (25th–50th) with palm length 10 cm (3rd–25th), fetal pads, mild tapering fingers, scoliosis. No abnormal gait (Figure 2A–E).

### 2.3. Case 2 (II:3): The Uncle

He was born at term, by spontaneous delivery complicated by fetal distress and use of forceps.

He achieved autonomous walking at one year, then he suffered a psychomotor development delay, mainly regarding language. In adolescence/adulthood, the subject presented a mild–moderate cognitive defect, a pleasant personality with behavioral problems, social interaction difficulties, and intolerance to stress/change. No seizures have been reported.

There were no peculiar health problems, except GE (gastroesophageal) reflux and constipation, strabismus, and amblyopia.

The main signs found in the last dysmorphology evaluation (at the age of 48 y) were height at 3rd–10th centile, OFC at 75th centile, facial dysmorphisms (brachycephaly, round face, high forehead, hypertelorism, arched eyebrows with synophrys, deviated nasal septum, bulbous nasal tip with the low hanging columella, lower lip not thick and only slightly everted), short hands with relative long fingers, mild joint stiffness (mainly elbow, interphalangeal joints). No scoliosis. No abnormal gait (Figure 2F–H).

### 2.4. Genetics

As part of the ID’s genetic workup, the following genetic tests have been performed over time with average results: peripheral blood high-resolution karyotype with subtelomeric FISH probes, FMR1 molecular analysis, and array-based Comparative Genomic Hybridization (aCGH). The reported presence of ID in the mother’s brother II:3 (although his facial dysmorphisms were significantly different to those observed in the proband), together with the health status of the mother, led us to consider the hypothesis of a common genetic etiology among the two affected males suggestive of an X-linked transmission model (Figure 1A).

The genetic analysis proceeded then with next-generation sequencing (NGS) panel of 71 genes at the Medical Genetics of the “IRCSS” Burlo Garofolo in Trieste.

A missense genetic variant c.566T > C; p.I189T was identified in exon 7 of the *RSK2* gene leading to substitution of an isoleucine for a tyrosine (I189T) in the proband (III:1). The subsequent segregation analysis performed on the mother (II:1) and the maternal uncle (II:3) confirmed the variant in both subjects.

The substitution identified involved a highly conserved amino acid across species, was predicted to be damaging by in silico tool [11], and was not present in publicly available databases such as dbSNP (build150) and gnomAD (http://gnomad.broadinstitute.org/, accessed on 10 March 2021). Furthermore, an analysis of the comparison of protein sequences demonstrates that isoleucine 189 was a highly conserved residue present in all known RSK proteins, suggesting its crucial functional role. Considering dominant transmission of the pathology and mother’s health status, we checked for a preferential X inactivation in leukocyte. X chromosome inactivation was based on differential methylation of the active and inactive X chromosome. (Two independent experiments were conducted with reproducible results). This analysis showed the presence of a skewed pattern (ratios > 83:17), suggesting preferential inactivation of the X-chromosome carrying the *RSK2* gene variant (Figure 1B).

Moreover, a mutation involving the same codon, c.566T > A; p.I189K, was once previously reported in two male siblings with a mild form of Coffin–Lowry syndrome (CLS) inherited from their healthy mother, but the functional impact was not investigated [3].

The patients described in the paper shared several features with our cases, such as hypotonia in childhood, delayed closing of fontanelles, cognitive defect (although in our cases the degree of severity is a bit higher), pleasant personality with behavioral anomalies (hyperactivity disorder, low tolerance to frustration, impulsiveness/outbursts of aggression), short stature, and macrocephaly (with onset in childhood). Typical facial dysmorphisms (high forehead, down slanting palpebral fissures, thick and everted lower lip, malar hypoplasia) were present in all patients except ours II:3. Tapered fingers were common in all cases. Neither the cases published, nor our patients experienced gait problems.

### 2.5. Functional Studies

**Figure 3:**
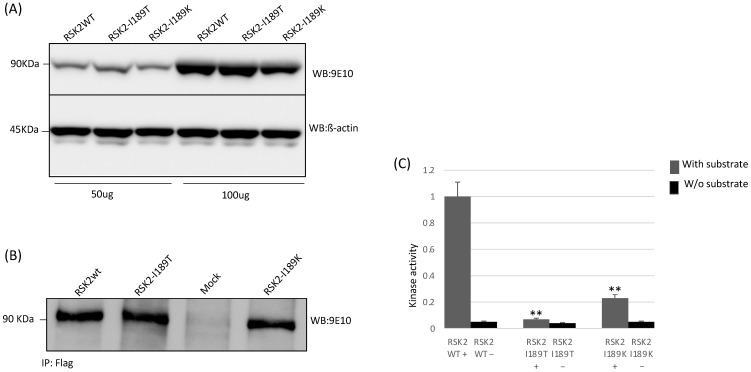
(**A**) Western blot analysis of transient transfection of cDNAs RSK2 wt., RSK2–I189T, RSK2–I189K, with 50 ug and 100 ug of loading proteins; (**B**) immunoprecipitation of equal proteins amount of wt., mutated proteins, and negative control (Mock); (**C**) measure of phosphotransferase activity from RSK2 wt., RSK2–I189T, RSK2–I189K proteins by kinase assay using S6 peptide, a standard RSK2 in vitro substrate. RSK2 was immunoprecipitated kinase assay with (+) and without (−) RSK2 substrate. Experiments were repeated three times with independently prepared cell extracts. ** *p* (value) < 0.001.

To better understand putative causal role of identified genetic variant, we conducted functional “in vitro” studies (protein translation analysis and kinase assay), for c.566T > C, p.I189T identified in our patient, and we included the c.566T > A p.I189K mutation already reported in literature but never functionally studied. Western blot analysis revealed standard expression and size of RSK2-I189T and RSK2-I189K proteins compared to wild type protein (Figure 3A). Since genetic variants were present in a protein kinase domain, we tested phosphorylation activity in both mutated proteins. The kinase assay showed that RSK2-I189T and RSK2-I189K had dramatically reduced kinase activity (Figure 3B,C).

## 3. Discussion

We described two related patients with an atypical CLS form inherited from their healthy mothers. CLS is an X-linked intellectual disability syndrome resulting from ribosomal S6 kinase 2 gene *(RSK2)* mutation. The two male patients showed intellectual disability (severe in the proband, mild/moderate in the uncle), hypotonia in infancy, late closing of the anterior fontanel, and short stature. Both had a pleasant personality and behavioral problems (hyperactivity disorder, low frustration tolerance, impulsiveness/outbursts of aggression). Facial dysmorphisms were typical in the proband and absent in the uncle. DNA analysis, performed by next-generation sequencing, identified a novel genetic variant c.566T > C; p.I189T in the *RSK2* gene and confirmed the CLS diagnosis. The variant was present in the N-terminal kinase domain. The same codon with a different aminoacidic exchange has already been described, c.566T > A; p.I189K, but this mutation’s biochemical effect has never been observed [3]. Patients’ publishing data showed mild MR, and similar personality and facial dysmorphism to our proband. Neither the cases published, nor our patients exhibit gait problems or significant progressive skeletal deformities.

## 4. Conclusions

For the first time, we conducted a functional study on the effect of both genetic variants identified. Our data showed a standard expression and size of RSK2-I189T and RSK2-I189K proteins compared to wild type. However, the S6 kinase assay revealed that RSK2-I189T and RSK2-I189K kinase activity were dramatically reduced. In conclusion, we detected a loss-of-function *RSK2* mutation with a decreased kinase activity in a three-generation family with an X-linked ID.

## 5. Material and Methods

### 5.1. Targeted Resequencing (TR)

We used a Targeted Resequencing (TR) panel including 71 genes involved in intellectual disabilities. These genes were selected according to data obtained from the most updated scientific literature and comprehensive public mutation databases such as HGMD (www.hgmd.org, accessed on 18 March 2021).

The TR panel ensured a total coverage of the targeted regions equal to 97.97% with 2188 pairs of multiplex-PCR primers, and the gene panel was defined using Ion Ampliseq Designer v1.2 (Life Technologies, Carlsbad, CA, USA). Targeted regions included only coding regions (CCDS) and 50 bp exons/introns boundaries of each of the 71 selected genes, spanning over 325.1 Kb.

According to the manufacturer’s protocols, DNA libraries were realized as described previously by P.Fontana et al. [12]. Sequencing data were analyzed following the Ion Torrent Suite^TM^ v5.2.2; Small Insertions and Deletions (INDELs) and Single Nucleotides Variations (SNVs) were pooled into a Variant Call Format (VCF) version 5.2.1.39 [13].

Human genome build-19 (hg19) was used as a reference. An Integrative Genomics Viewer (IGV) was adopted to confirm the variant calls and visualize the read alignment [14].

To annotate INDELS and SNVs, we employed the software ANNOVAR [15]. The DNA variants identified were further confirmed by direct bidirectional Sanger sequencing on an ABI PRISM 3500Dx Genetic Analyzer sequencer (Life Technologies, Carlsbad, CA, USA), ABI PRISM 3.1 Big Dye terminator chemistry (Life Technologies, Carlsbad, CA, USA) according to the manufacturer’s instructions [16].

### 5.2. X-Chromosome Inactivation (XCI)

For the analysis, 500 ng of genomic DNA from the proband (III:1) and from the proband’s mother (II:1) was digested as described above and incubated at 95 °C for 10 min. 50 ng of digested and undigested DNA was amplified according to the protocol of a previous study [3] for amplification of the human androgen receptor (HAR) gene. Since this fragment has two HpaII restriction enzyme sites, only methylated DNA was amplified.

### 5.3. DNA Cloning

The wild-type (wt.) and mutant human *RSK2* (RSK2-I189T) (RSK2-I189K) cDNAs were cloned into a pCMV6-Entry vector (by Origene, Rockville, MD, USA), Myc tagged. For transfection the vectors were further purified through the Plasmid Midi Kit, (Qiagen, Hilden, Germany).

### 5.4. Cell Culture and Transient Transfection

HEK293 cells were cultured in Dulbecco’s Modified Eagle Medium (DMEM) containing 1 g/L glucose supplemented with 10% fetal calf serum (FCS), penicillin (100 U/mL) and streptomycin (0.1 mg/mL). 5 × 10^5^ HEK293 cells were seated in 6 cm plate and incubated overnight. Cells were transfected using the standard Calcium Phosphate Transfection Method. according to the protocol of a previous study by di Stazio et al. [17]. Cells were collected 48 h after transient transfection.

### 5.5. Western Blot Analysis

For protein analysis, HEK293 cells were maintained using DMEM supplemented with 10% FBS, and penicillin-streptomycin (Invitrogen LifeTechnologies, Carlsbad, CA, USA) 

Protein levels for RSK2 were assessed by Western blot. After incubation, cells were lysed with IPLS buffer (50 mM Tris-HCL pH7.5, 120 mM NaCl, 0.5 mM EDTA and 0.5% Nonidet P-40) containing protease inhibitors (Roche, Indianapolis, IN, USA).

After sonication and pre-clearing, protein concentrations were determined by Bradford Assay (Biorad, Hercules, CA, USA). The proteins were resolved on 10% SDS-PAGE and afterwards transferred to nitrocellulose membranes. Immunoblots were blocked in 5% BSA (Roche) in TBST (50 mM Tris-HCl, pH 7.4, 200 mM NaCl, and 0.1% Tween 20). Membranes were then incubated with the primary antibodies against the Myc epitope (Sigma, St. Louis, MO, USA) and ß-Actin antibody (Sigma, St. Louis, MO, USA) and the appropriate secondary antibodies. Proteins were detected with the ECL detection kit (GE Health Care Bio-Sciences, Princeton, NJ, USA) [18].

### 5.6. Immunoprecipitation and Protein Kinase Assay

The immunoprecipitation of wt. and mutated proteins was performed using an Anti-DDK Agarose Immunoprecipitation Kit (OriGene, Rockville, MD, USA). The same immunoprecipitation has been divided into 3 aliquots. One was used for Western blot quantification, other parts for kinase assay with and without specific substrate. According to the manufacturer’s instructions, kinase activity was measured using the ADP-GLO assay system (Promega, Madison, WI, USA).

## Data Availability

All data generated or analyzed during this study are included in this published article.

## Abbreviations

CLS	Coffin–Lowry Syndrome
ID	Intellectual disability

## References

[1] Maystadt I., Destrée A., Benoit V., Aeby A., Lederer D., Moortgat S., Jurkiewicz D., Krajewska-Walasek M., Hanauer A., Thomas G. (2013). RSK2mutation co-segregates with X-linked intellectual disability and attenuated Coffin-Lowry phenotype in a three-generation family. Clin. Genet..

[2] Lowry B., Miller J.R., Fraser F.C. (1971). A New Dominant Gene Mental Retardation Syndrome. Am. J. Dis. Child..

[3] Manouvrier-Hanu S., Amiel J., Jacquot S., Merienne K., Moerman A., Coeslier A., Labarriere F., Vallee L., Croquette M.F., Hanauer A. (1999). Unreported RSK2 missense mutation in two male sibs with an unusually mild form of Coffin-Lowry syndrome. J. Med. Genet..

[4] Temtamy S.A., Miller J.D., Hussels-Maumenee I. (1975). The Coffin-Lowry syndrome: An inherited faciodigital mental retardation syndrome. J. Pediatr..

[5] Hanauer A., Young I.D. (2002). Coffin-Lowry syndrome: Clinical and molecular features. J. Med. Genet..

[6] Pereira P.M., Schneider A., Pannetier S., Heron D., Hanauer A. (2009). Coffin–Lowry syndrome. Eur. J. Hum. Genet..

[7] Ikuta M., Kornienko M., Byrne N., Reid J.C., Mizuarai S., Kotani H., Munshi S.K. (2007). Crystal structures of the N-terminal kinase domain of human RSK1 bound to three different ligands: Implica-tions for the design of RSK1 specific inhibitors. Protein Sci..

[8] Turner G., Lower K., White S., Delatycki M., Lampe A., Wright M., Smith J.C., Kerr B., Schelley S., Hoyme H. (2004). The clinical picture of the Börjeson-Forssman-Lehmann syndrome in males and heterozygous females with PHF6 mutations. Clin. Genet..

[9] Merienne K., Jacquot S., Pannetier S., Zeniou M., Bankier A., Gecz J., Mandel J.L., Mulley J.C., Sassone-Corsi P., Hanauer A. (1999). A missense mutation in RPS6KA3 (RSK2) responsible for non-specific mental retardation. Nat. Genet..

[10] Field M., Tarpey P., Boyle J., Edkins S., Goodship J., Luo Y., Moon J., Teague J., Stratton M.R., A Futreal P. (2006). Mutations in the RSK2(RPS6KA3) gene cause Coffin-Lowry syndrome and nonsyndromic X-linked mental retardation. Clin. Genet..

[11] Zhang M., Huang C., Wang Z., Lv H., Li X. (2020). In silico analysis of non-synonymous single nucleotide polymorphisms (nsSNPs) in the human GJA3 gene associated with congenital cataract. BMC Mol. Cell Biol..

[12] Fontana P., Morgutti M., Pecile V., Lenarduzzi S., Cappellani S., Falco M., Scarano F., Lonardo F. (2017). A novel OTOA mutation in an Italian family with hearing loss. Gene Rep..

[13] Danecek P., Auton A., Abecasis G., Albers C.A., Banks E., De Pristo M.A., Handsaker R.E., Lunter G., Marth G.T., Sherry S.T. (2011). The variant call format and VCFtools. Bioinformatics.

[14] Lenarduzzi S., Morgan A., Faletra F., Cappellani S., Morgutti M., Mezzavilla M., Peruzzi A., Ghiselli S., Ambrosetti U., Graziano C. (2019). Next generation sequencing study in a cohort of Italian patients with syndromic hearing loss. Hear. Res..

[15] Wang K., Li M., Hakonarson H. (2010). ANNOVAR: Functional annotation of genetic variants from high-throughput sequencing data. Nucleic Acids Res..

[16] Home|Research Square. https://www.researchsquare.com/.

[17] Di Stazio M., Collesi C., Vozzi D., Liu W., Myers M., Morgan A., D’adamo P.A., Girotto G., Rubinato E., Giacca M. (2019). TBL1Y: A new gene involved in syndromic hearing loss. Eur. J. Hum. Genet..

[18] Di Stazio M., Morgana A., Brumat M., Bassani S., Dell’Orco D., Marino V., Garagnani P., Giuliani C., Girotto P.G.G. (2020). New age-related hearing loss candidate genes in humans: An ongoing challenge. Gene.

